# A Fluorescent Conjugated Polar Polymer for Probing Charge Injection in Multilayer Organic Light-Emitting Transistors

**DOI:** 10.3390/molecules29143295

**Published:** 2024-07-12

**Authors:** Salvatore Moschetto, Benedetta Maria Squeo, Francesco Reginato, Mario Prosa, Mariacecilia Pasini, Stefano Toffanin

**Affiliations:** 1 Institute of Nanostructured Materials (ISMN), National Research Council (CNR), Via P. Gobetti 101, 40129 Bologna, Italy; francesco.reginato@ismn.cnr.it (F.R.); mario.prosa@cnr.it (M.P.); 2 Institute of Chemical Sciences and Technologies “G. Natta” (SCITEC), National Research Council (CNR), via Corti 12, 20133 Milan, Italy; benedetta.squeo@scitec.cnr.it (B.M.S.); mariacecilia.pasini@scitec.cnr.it (M.P.)

**Keywords:** organic light-emitting transistors, conjugated polar polymer, heterostructure, ambipolar charge transport, functional interfaces

## Abstract

Ambipolar organic light-emitting transistors (OLETs) are extremely appealing devices for applications from sensing to communication and display realization due to their inherent capability of coupling switching and light-emitting features. However, their limited external quantum efficiency (EQE) and brightness under ambipolar bias conditions hamper the progress of OLET technology. In this context, it was recently demonstrated in multi-stacked devices that the engineering of the interface between the topmost electron-transporting organic semiconductor (e-OS) and the emission layer (EML) is crucial in optimizing the recombination of the minority charges (i.e., electrons) and to enhance EQE and brightness. Here, we introduce a new light-emitting conjugated polar polymer (CPP) in a multi-stacked OLET to improve the electron injection from e-OS to EML and to study, simultaneously, electroluminescence-related processes such as exciton formation and quenching processes. Interestingly, we observed that the highly polar groups present in the conjugate polymer induced polarization-related relevant charge-trapping phenomena with consequent modulation of the entire electrostatic field distribution and unexpected optoelectronic features. In view of the extensive use of CPPs in OLETs, the use of multifunctional CPPs for probing photophysical processes at the functional interfaces in stacked devices may speed up the improvement of the light-emission properties in OLETs.

## 1. Introduction

Organic light-emitting transistors (OLETs) have been widely recognized as promising alternative devices to the well-known organic light-emitting diodes (OLEDs) [1,2] since they are expected to overcome some limitations inherent in vertical stack architecture [3,4]. In fact, despite their wide use in consumer electronics, OLEDs may show limited performance due to reduced electrode transparency [5,6] or optical cavity [7,8] effect because the photons, once generated, are confined between two stacked electrodes and forced to outcouple passing through at least one electrode. On the other hand, OLETs [9,10,11] are planar devices capable of integrating the logical switching functionality of the transistor with light emission. Well-balanced ambipolar OLETs [12] enable the recombination of opposite charges horizontally within the channel, thus generating a light-emitting stripe as large as a few tens of micrometers. These features guarantee not only the emission of light far away from the charge-injecting/-collecting electrodes [13,14,15], but also the realization of an intrinsically microscaled light source.

In order to allow the shifting of the light-emitting stripe within the channel, it is mandatory that both electrons and holes are efficiently transported in the active semiconducting layer(s) [16]. In this context, the availability of efficient electron-transporting organic semiconductors (OSs) in comparison with hole-transporting compounds is very limited in organic field-effect transistors [17,18,19]. Several strategies have been implemented to overcome this limitation and thus to guarantee efficient light emission [20], including the use of innovative materials, such as multifunctional organic semiconductors [21,22,23], innovative transistor layouts such as those with double, split- and overlapping gates [24], and multilayer stacked configurations, such as bilayer and trilayer ones [15,25]. In particular, in the case of unipolar multilayer stacked devices where only one type of OS is present (typically hole-transporting OS, i.e., h-OS) the charge recombination and the consequent exciton formation and light emission are located in the emissive layer (EML) in the proximity of the electrode collecting the majority charge carriers (i.e., drain electrode) [26,27].

In recent years, conjugated polar polymers (CPPs) have been effectively introduced as electron-injection layers (EILs) in OLEDs [28,29,30] and unipolar multilayer OLETs [31]. Solution-processable CPPs are π-conjugated polymers endowed with polar nonionic side groups [32], while their ionic counterparts are conjugated polyelectrolytes (CPEs) [33,34,35,36]. Even though CPEs can be used as effective EIL in polymer LED (PLEDs) [37,38,39,40], dipole formation correlated to ion migration in biased devices might increase the quenching of excitons in time [41]. Indeed, we have recently demonstrated that the use of a CPE such as PFN^+^Br^−^ in OLET devices negatively affects the performance in terms of EL intensity and efficiency. In particular, we demonstrated that the use of CPP compounds may increase the light power and EQE [42,43] by a factor of 2 and 5, respectively, in comparison to the use of the same CPE counterpart.

Considering multilayer light-emitting transistor devices with h-OS at the interface with the dielectric, it is mandatory to probe and optimize the interface between the (outermost) electron-transport OS (e-OS) layer and the EML layer, whose capability of emitting efficiently electroluminescence strictly depends on the injection and transport of the minority charge carriers (i.e., electrons).

In our recent paper, we demonstrated in multilayer OLET that the engineering of the interface between the topmost electron-transport OS layer located under the electrodes and the EML is pivotal for improving the percolation of electrons (minority charge carrier) into the EML to improve balanced charge recombination [44]. Specifically, we inserted and optimized a well-known electron-injection material between the e-OS and the EML to improve the exciton formation. In this scenario, the introduction of a CPP material between e-OS and the EML might modulate the interfacial energy and/or the alignment of local energy levels [45], eventually improving the electron accumulation inside the e-OS and/or the injection in the EML. 

**Figure 1:**
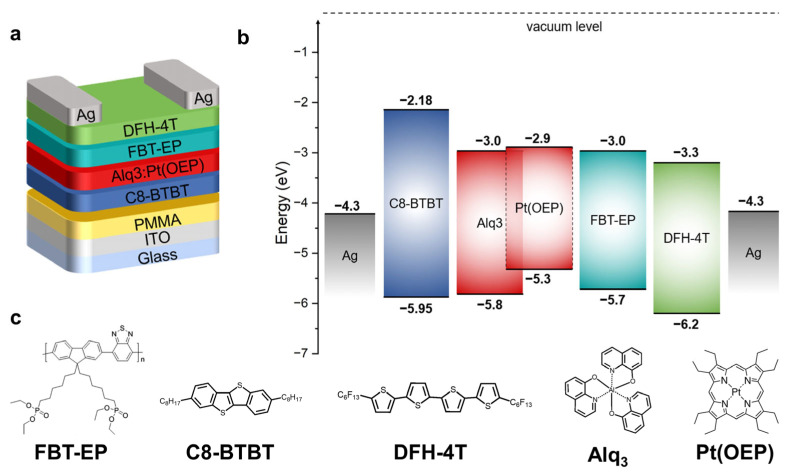
(**a**) Device structure of h-OS/EML/e-OS OLET comprising FBT-EP as interlayer (the reference device does not include it). (**b**) Energy level and (**c**) chemical structures of all employed materials in the h-OS/EML/e-OS OLET device.

In this paper, we introduce a multifunctional CPP, poly [9,9-bis(6′-diethoxylphosphorylhexyl)-alt-benzothiadiazole] (FBT-EP, whose chemical structure is reported in Figure 1a), which has been successfully tested in OLEDs and organic solar cells (OSCs), at the interface between an e-OS and an EML in a unipolar multilayer OLET [46].

FBT-EP is a neutral alternating copolymer based on fluorene and benzothiadiazole with pendant phosphonate groups, which confer water–alcohol solubility and improve electron injection thanks to the favorable interfacial dipole and the coordination interaction of the phosphonate groups with the metal electrodes [46,47,48,49,50].

Interestingly, the FBT-EP shows relevant emissive properties [46] and might be considered an optical probe for studying electroluminescence-related processes such as exciton formation and quenching processes once deposited on top of the EML in multilayer field-effect transistors. It should be highlighted that the solubility of FBT-EP in polar solvents such as ethyl alcohol enables the smooth deposition as thin films in stacked structures composed of organic molecular materials that are typically soluble in organic solvents and deposited by vacuum sublimation. 

In particular, FBT-EP was inserted at the e-OS/EML interface of a multilayer OLET composed of (i) 2,7-dioctyl [1]benzothieno [3,2-b] [1] benzothiophene (C8-BTBT) as the h-OS, (ii) tris(8-hydroxyquinoline)aluminum(III) (Alq_3_) doped with a red emitter platinum-octaethylporphyrin Pt(OEP) as the host-guest EML, and (iii) α, ω-diperfluorohexyl-4T (DFH-4T) as e-OS.

Despite its optimal energy level alignment with the other organic semiconductors and emissive dye in the multilayer stack, as reported in Figure 1b,c, the introduction of FBT-EP in the active region of the transistor showed unexpected optical and electrical responses under operational bias conditions. Indeed, we observed a reduction in the optical power of the device with respect to the reference device without FBT-EP as an interlayer.

We implemented a very comprehensive approach in the investigation of the effect of the introduction of a luminescent CPP layer into field-effect device architecture by fabricating devices comprising FBT-EP as the single active layer and at the interface with the other functional layers in the transistor (i.e., electron-injecting electrode, dielectric, EML, e-OS).

We observed that high dipole-moment moieties such as the ones present in conjugated polar polymers may modulate charge accumulation at relevant functional interfaces in multilayer field-effect transistors due to the possible charge trapping once the device is biased, with consequent re-modulation of the entire electrostatic field distribution within the device active region.

Considering the increasing implementation of CPP materials in multilayer devices, it is mandatory to proceed in a systematic rationalization of the (also undesired) effects in the light-formation processes when charge- and/or electric field-induced processes are activated in highly polarized and polarizable thin films present in multilayer field-effect transistors.

## 2. Results and Discussion

All OLETs were fabricated on a transparent substrate composed of glass/indium titanium oxide (ITO)/poly(methyl methacrylate) (PMMA) to promote the downward collection of luminescence, where ITO and PMMA are the gate electrode and the dielectric layer, respectively. All organic layers of h-OS/EML/e-OS OLET were deposited by thermal evaporation. Consecutively, the following were deposited on top of the PMMA layer: (i) C8-BTBT [51] as the h-OS, (ii) Alq_3_ doped with a red emitter Pt(OEP) [52,53] as the host-guest EML, and (iii) DFH-4T [54] as e-OS.

In the prototypal device structure, as reported in Figure 1a, we introduced firstly a 10 nm thick FBT-EP layer at the interface between DFH-4T and EML as an electron-injecting layer by exploiting the effective deposition by spin-coating in an alcoholic solution. The chemical structures and the relative energy levels of the compounds used in this multilayer OLET are depicted, respectively, in Figure 1b,c.

From the energy level diagram, it is possible to note the excellent energy level alignment of the highest occupied molecular orbital (HOMO) of the C8-BTBT with the HOMO of the matrix Alq_3_, as well as the low unoccupied molecular orbital (LUMO) of the DFH-4T with respect to that of Alq_3_. The choice of h-OS and e-OS with low energy barriers toward the HOMO/LUMO (respectively) of the matrix guarantees, therefore, both efficient hole- and electron-injection into the EML. Moreover, it is possible to observe that the LUMO level of FBT-EP is perfectly aligned with that of Alq_3_, thus avoiding any energy barrier for the electron injection from DFH-4T toward the matrix even when FBT-EP is present. In this framework, we might exclude any hindrance due to energy misalignment in the exciton formation when FBT-EP is present, while instead considering the modulation of the OLET emissive properties only in relation to the polarization effects due to the CPP material.

**Figure 2:**
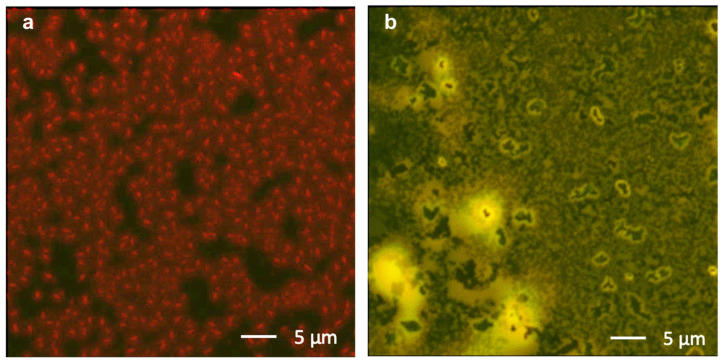
CLSM images of the active region within the channel of the OLETs presenting (**a**) h-OS/EML and (**b**) h-OS/EML/FBT-EP. The excitation laser wavelengths are 405 nm in (**a**) and 488 nm in (**b**), while the PL signal is collected in the spectral range higher than 600 nm (red channel) in (**a**,**b**) in the spectral range of 488–543 nm (green channel) only in (**b**).

As a preliminary step, we checked if the processing in an alcoholic solution of the FBT-EP might guarantee the suitable homogenous coverage of the deposited film on top of the EML. In Figure 2, we report Confocal Laser Scanning Microscope (CLSM) images of the channel regions of the h-OS/EML/FBT-EP transistor: by selectively collecting the photoluminescence (PL) signal from the EML (Figure 2a) and from the FBT-EP layer (Figure 2b) within the same single device by using two different channels of the detector, it is possible to obtain morphological information of the buried interface [42]. In particular, it can be observed that the granular morphology of the EML (which can be related to the possible aggregation of the dye moieties in bright-red micrometric domains) is likely replicated by the superimposed FBT-EP layer, thus providing a thick, compact, yellowish-emitting film. It is worth mentioning that the EML has no residual emission in the green range of the CLSM photodetector after the complete energy transfer from the Alq_3_ matrix and the Pt(OEP) dyes [55].

### FBT-EP in h-OS/EML/e-OS OLET

**Figure 3:**
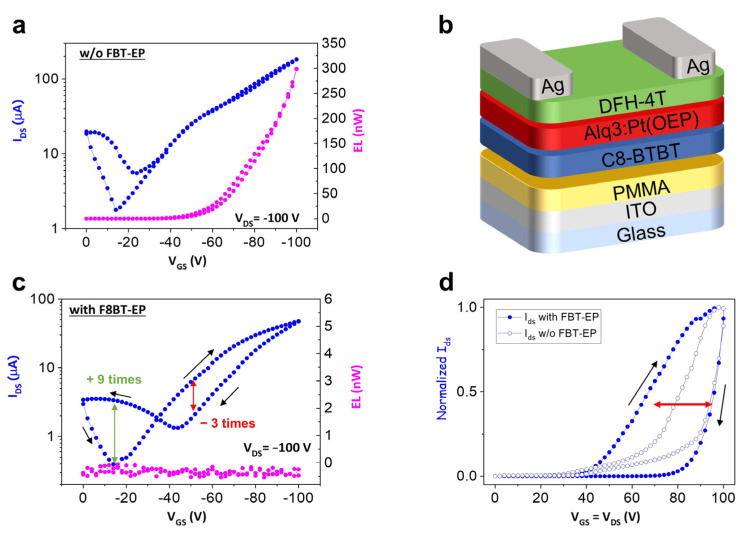
P-type transfer curve in saturation regime (blue curves) and related electroluminescence curves (pink curves) of (**a**) reference h-OS/EML/e-OS device and (**c**) h-OS/EML/EIL/e-OS device containing FBT-EP. The right and left pointing arrows represent the onward and backward directions of measurement, respectively. The double arrows in green and red highlight the amplitude of the hysteresis. (**b**) Schematic representation of the reference device comprising h-OS/EML/e-OS. (**d**) Normalized n-type locus curve of h-OS/EML/e-OS OLET with and without FBT-EP (filled and empty dot curves, respectively).

The p-polarized transfer curve of the reference h-OS/EML/e-OS device, which is schematized in Figure 3b, in the saturation regime (Figure 3a) shows a V-shaped curve [56]. In particular, by comparing the left and right branches of the curve, it is evident that the lateral hole and electron transport are not well balanced and the hole transport is predominant with hole mobility (µ_h_) of 7.85 × 10^−2^ cm^2^ V^−1^ s^−1^. As a further confirmation, we observed remarkable light emission only when the device was p-polarized (Figure 3a). Following the introduction of FBT-EP between the EML and e-OS layers, the behavior of the device was largely modified, as highlighted by the I_DS_ saturation transfer curve, and, most of all, no light emission was observed (Figure 3c). Considering the I_DS_ current curve of Figure 3c, when the gate bias swipes from 0 toward more negative voltage values, the I_DS_ curve follows the expected trend as in the h-OS/EML/e-OS reference device. Once it reaches the saturation condition at V_DS_ value at −100 V, the reverse sweep of V_GS_ is correlated to a significant hysteresis of the I_DS_ current. In particular, at around −50 V of V_GS_ (i.e., formally, at ambipolar bias conditions being V_DS_ equal to −100 V), the collected I_DS_ is 3 times lower than the corresponding value in the forward direction. Sweeping from −50 V to 0 V (formally, electron injection and accumulation mode in the e-OS layer), the I_DS_ is higher with respect to the corresponding one collected at forward bias, specifically reaching a nine-times-higher value. 

Comparing the devices with and without the FBT-EP layer, the collected source-drain current at saturation p-polarized bias conditions is decreased by an order of magnitude when the interlayer is introduced (from 182 to 47 µA at −100 V of applied bias): this evidence might indicate that the holes injected at the source electrode are not completely collected at the drain electrode, but they are rather blocked within the interlayer, giving rise to hysteresis in the transfer curve. Moreover, considering the larger hysteresis loop in the saturation transfer characteristic reported in Figure 3c in the bias range V_GS_ < |50 V|, it is possible to speculate that the increased contribution in the electron current in the backward branch of the I_DS_ curve (as the applied V_GS_ bias becomes more positive), so that mainly electrons should be transported, is related to the collection at the source electrode of the electrons de-trapped from the FBT-EP/DFH-4T interface or the bulk of the FBT-EP layer, which had been previously accumulated during the onward bias corresponding to the transport in the channel and collection at the drain electrode of the holes (which, in turn, corresponds to the injection of electrodes from the drain electrode).

To estimate the entity of the possible charge trapping process promoted by FBT-EP in the multilayer stack transistor where opposite charges are injected and typically trapped in the EML for generating excitons, we fabricated three transistor architectures in simplified configuration by introducing the FBT-EP layer to probe the relevant interfaces of the multilayer stack transistor. In particular, the prototypal devices comprised (i) C8-BTBT/FBT-EP (h-OS/EIL) in order to investigate the modulation of the injection of holes from the source electrode to the h-OS in the presence of the CPP compound (Figure S1a); (ii) C8-BTBT/Alq_3_:Pt(OEP) (h-OS/EML) to reproduce the h-OS/EML device sub-architecture, and (iii) C8-BTBT/Alq_3_:Pt(OEP)/FBT-EP (h-OS/EML/EIL) in order to investigate the capability of injection of electrons into the EML of the CPP compound (Figure S1c). For both prototypal devices, the FBT-EP layer was 10 nm thick, as it was in the complete multilayer stack OLET, and a reference device for comparing the optoelectronic performance of the C8-BTBT/Alq_3_:Pt(OEP)—i.e., h-OS/EML—device was considered (Figure S1b).

When considering h-OS/EIL and h-OS/EML devices, the p-polarized saturation transfer curves show very similar behavior (and thus for the two layers) in terms of electrical parameters and hysteresis. In fact, the extracted maximum I_DS_ and calculated µ_h_ are comparable, such as 205 µA and 1.06 × 10^−1^ cm^2^ for V^−1^ s^−1^ for the h-OS/EIL device and 235 µA and 1.28 × 10^−1^ cm^2^ V^−1^ s^−1^ for the h-OS/EML device. Both curves show non-negligible but limited hysteresis. Consequently, we can infer that no remarkable inhibition of hole injection into h-OS is expected at the FBT-EP/source electrode interface. Considering the h-OS/EML/EIL device (Figure S1c), the maximum I_DS_ and the mobility are in line with both h-OS/EML and h-OS/EIL devices (so that, 223 µA and 1.34 × 10^−1^ cm^2^ V^−1^ s^−1^, respectively). Surprisingly, despite the comparable electrical characteristics of h-OS/EML and h-OS/EML/EIL, light emission is suppressed when FBT-EP is inserted in the h-OS/EML/EIL (Figure S1b), as in the case of the multilayer stack h-OS/EML/EIL/e-OS device (Figure 3b, pink curve). Hence, the electroluminescence signal (either because of exciton quenching or inhibited exciton formation) is affected by the electron injection and transport at the drain electrode when FBT-EP is introduced in the transistor. 

In order to further analyze the behavior of the ambipolar multilayer h-OS/EML/EIL/e-OS transistor, we investigated the n-type unipolar characteristics by performing an n-polarized locus curve [15,57], by sweeping simultaneously the V_DS_ and V_GS_ bias and keeping V_DS_ = V_GS_. (Figure 3d). When the FBT-EP layer is present in the device, a larger hysteresis in the I_DS_ current can be observed. The locus curves of the multilayer OLETs with and without the FBT-EP layer were normalized to their own maximum values of I_DS_ in order to compare properly the hysteresis loops: in this regard, it must be noticed that I_DS_ maximum is reduced by almost a factor of five in the case of FBT-EP.

Considering that in the p-saturation regime, the electrons were injected from the drain electrode of the h-OS/EML/EIL/e-OS device (Figure 3d and Figure 3c, respectively), the larger hysteresis loop and the limited I_DS_ value collected in the locus characteristic and in the p-polarized transfer curve (in saturation regime) suggest that (i) the electrons could be entrapped within the FBT-EP layer or at the FBT-EP/DFH-4T interface with consequent inhibition of exciton formation in the EML and/or (ii) the LUMO level of FBT-EP may become higher in energy due to the intrinsic polarizability of FBT-EP with consequent formation of electric dipoles that block the electron injection. Electric dipole formation at the interfaces can be due to high-polarizing materials [58] or by interaction with other molecules, such as Alq_3_ or DFH-4T, which leads to spontaneous orientation polarization (SOP) that, in turn, strongly affects the external quantum efficiency (EQE) of the device [59,60]. Moreover, the absence of light in both the devices presenting FBT-EP, i.e., h-OS/EML/EIL and h-OS/EML/EIL/e-OS devices, regardless of the decrease in the collected I_DS_ current, suggests also the possibility of a recombination zone displacement in the axial direction through the multilayer stack from a region within the EML to a region within the FBT-EP layer.

In order to better investigate the role of FBT-EP in quenching the electroluminescence signal of the OLET, the replacement of the Alq_3_:Pt(OEP) emission layer with an FBT-EP layer of the same thickness (i.e., 30 nm) could be an alternative strategy. Indeed, since the substitution with phosphonate groups does not drastically alter the emissive properties of the polymers, as reported for PF-EP [61], FBT-EP also possesses good emissive properties and can be used as an active material (emissive layer) in OLEDs. Accordingly, FBT-EP was inserted in a suitable device architecture, such as an OLED device comprised by ITO/PEDOT:PSS/PVK/FBT-EP/TPBi/LiF/Al (whose layers stack and correlated-material energy levels are reported in Figure S2a). The I-V current curve of the fabricated device (blue line, Figure S2b) shows the typical exponential behavior of a diode, and the corresponding curve related to the electroluminescence signal (green line, Figure S2b) shows a maximum light emission at higher bias values, as expected for a similar structure already reported in the literature [46].

**Figure 4:**
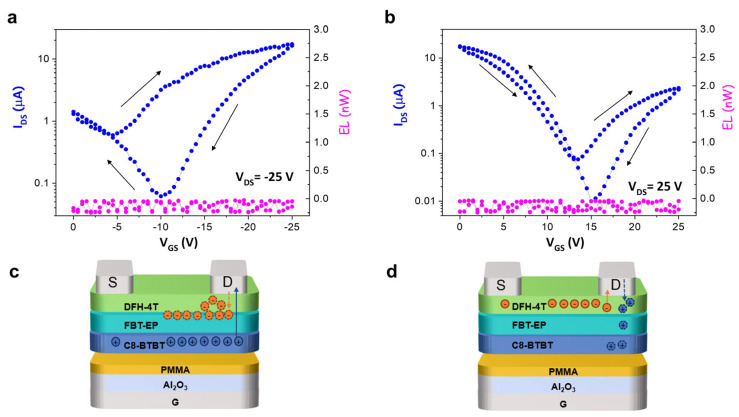
P-type (**a**) and n-type (**b**) transfer curves in saturation regime (blue curves) of a h-OS/FBT-EP/e-OS device with its related (**c**) p-type and (**d**) n-type schematic operation. The right and left pointing arrows represent the onward and backward directions of measurement, respectively.

As a result, the FBT-EP layer was used as the emissive layer in a h-OS/EML/e-OS devices (Figure 4). It is worth mentioning that, since the possible modulation of the polarizability of FBT-EP is correlated to the effective electric field experienced by the organic stack, we replaced the PMMA dielectric single-layer with a high-k dielectric heterostructure composed of aluminum oxide (Al_2_O_3_, whose layer thickness was 35 nm) and PMMA (which is used for planarizing the oxide surface and enabling the proper growth of the h-OS and whose layer thickness was 20 nm). While the saturation regime in a device with a fully PMMA dielectric single-layer was reached at −100 V of V_GS_ (with V_DS_ at −100 V), by using an Al_2_O_3_-based dielectric, the saturation regime was reached at −25 V of V_GS_ (with V_DS_ at −25 V), thus reducing the transveral electrostatic potential along the channel by a factor of three by considering the space-charge-induced potential defined in reference [62]. So, the fabricated device was ITO/Al_2_O_3_-PMMA/C8-BTBT/FBT-EP/DFH-4T/Ag, where DFH-4T was used as an electron-injecting and -transporting layer. Even though quite well-balanced ambipolar charge transport is evident by both the p- and n-polarized transfer curves, still no light emission was collected, with consequent evidence that the radiative charge recombination processes were not largely limited by the electrostatic potential drop at the drain.

If we consider in more detail the p-polarized saturation transfer curve of the h-OS/FBT-EP/e-OS device reported in Figure 4a, it is possible to better describe the process on the basis of the inhibited exciton formation in FBT-EP. As sketched in Figure 4c, the holes extracted at the drain electrode in p-polarized saturation bias condition had to pass through the FBT-EP layer and had been blocked at the DFH-4T/FBT-EP interface, resulting in a pronounced hysteresis when the device was backward-biased: indeed, the minor I_DS_ value at a V_GS_ of −10 V in the back direction with respect to the forward direction could indicate that electrons accumulated at the DFH-4T/FBT-EP interface. We do not exclude that this effect is also helped by the slight energy mismatch between the HOMO levels of DFH-4T and FBT-EP. It is likely that the trapping of holes happens in the FBT-EP layer at the very interface with DFH-4T since (in backward-bias) injected electrons are now trapped at that interface and not capable of diffusing in FBT-EP to recombine with holes. Probably, a further contribution to the reduction in the collected I_DS_ is given by an internal electric field generated by the charge distribution of fixed charges in FBT-EP that opposes the hole transport through the stack. 

When the device was subjected to a saturation transfer characteristic in n-polarization, electrons formed a channel in DFH-4T at the interface with FBT-EP when increasing the V_GS_ bias: when collected at the drain electrode in saturation condition, electrons were less subjected to the competing trapping induced by the as-blocked (fewer) holes in the FBT-EP layer, with consequent less-pronounced hysteresis. (Figure 4b).

Finally, the absence of light in the h-OS/FBT-EP/e-OS structure demonstrates the absence of recombination of charges in FBT-EP layer because of the absence of minority charges. At this point, considering that (i) in both the structures where FBT-EP is interfaced with metal electrode and organic semiconductor layer, i.e., h-OS/EML/EIL and h-OS/EML/EIL/e-OS, respectively, the charge recombination is inhibited, (ii) the presence of a thicker of FBT-EP film as EML does not enable the electroluminescence emission, and (iii) the hysteresis loop is extremely enlarged in device containing FBT-EP (locus curve, Figure 3d), it is possible to assert that the LUMO level of FBT-EP is likely modulated by local electrical dipole formation and/or orientation at the organic/hybrid interfaces in the region next to the drain electrode. In this context, when an e-OS layer is deposited on top of FBT-EP (which enables, in principle, the accumulation of electrons at the interface with FBT-EP), the reduction in I_DS_ collected at drain electrodes with respect to the corresponding devices without FBT-EP clearly highlights that electrons cannot percolate in the EML while being blocked within the e-OS.

**Figure 5:**
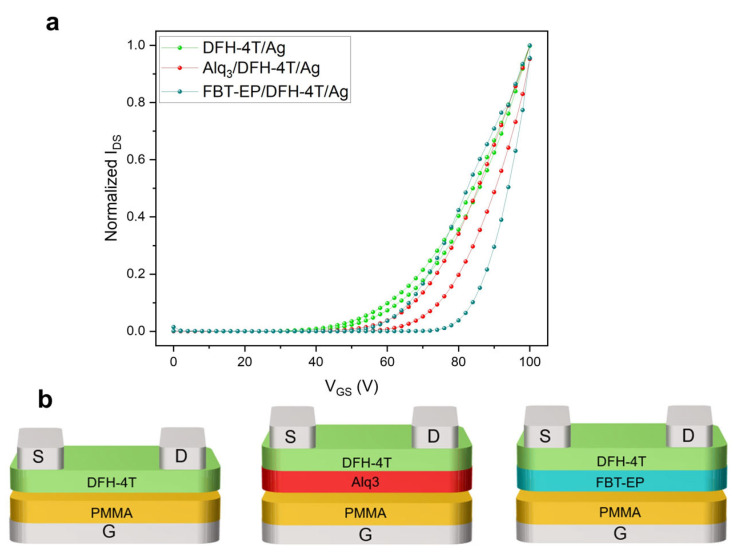
(**a**) Normalized n-type saturation curves of DFH-4T/Ag (green curve), Alq_3_/DFH-4t/Ag (red curve) and FBT-EP/DFH-4t/Ag (blue curve) devices and (**b**) their relative structures, respectively.

In order to better clarify the role of the interface between the electron-transporting semiconductor and CPP compound in quenching the electroluminescence in multilayer ambipolar OLETs, we fabricated transistors based on sub-systems of the entire multilayer OLETs comprising only e-OS for lateral charge transport, where (i) DFH-4T is the only active layer in direct contact with the dielectric layer (i.e., glass/ITO/PMMA/DFH-4T/Ag), (i) DFH-4T is deposited on top of the host material of the luminescent host–guest system used as standard EML (i.t. glass/ITO/PMMA/Alq_3_/DFH-4T/Ag), and (iii) DFH-4T deposited on top of FBT-EP conjugated polar polymer (i.e., glass/ITO/PMMA/FBT-EP/DFH-4T/Ag). The schematics of the devices are reported in Figure 5: in the case of the bilayer-based transistor, the layer in which electrons are expected to percolate is deposited directly onto the dielectric layer in order to facilitate the investigation of the polarizability when the device is biased and the electrons are accumulated at the interface with DFH-4T.

In Figure 5a, we report n-polarized saturation transfer characteristics that were normalized at their own source-drain current maximum value. Considering the I_DS_ curve of the device with a DFH-4T layer in direct contact with the PMMA dielectric as a reference since no electron percolation in the dielectric layer was expected (green line in Figure 5a), we observed an increasing hysteresis in the collected I_DS_ curves (forward and backward bias) passing from the transistor with Alq_3_ (red curve) and FBT-EP (blue curve) in direct contact with the dielectric layer.

From the electrical characteristics, we infer that the polarizability of the FBT-EP layer is likely pivotal in trapping electrons at the interface between the accumulation layer of the e-OS and the underneath CPP layer: indeed, the collected electron current dropped from 900 µA to 1.5 µA from DFH-4T/PMMA to DFH-4T/FBT-EP interfaces. Interestingly we may observe that the Alq_3_ host matrix is also polarizable, as demonstrated by the non-negligible current hysteresis loop in the curve [60]; however, the capability of trapping electrons of Alq_3_ is less extended than FBT-EP. In addition, the increase in threshold gate voltages of 45.2, 56.5, and 66.5 V is noticed passing, respectively, from the DFH-4T/PMMA, to DFH-4T/Alq_3_ to DFH-4T/FBT-EP interfaces, indicating that in the latter interface, more electrons are likely needed to fill the traps and permit the current flow within the channel. This observation confirms the capability of FBT-EP polymer to trap electrons when used in multilayer transistor device configuration.

Considering that Alq_3_ and FBT-EP present the same HOMO and LUMO levels (at least the energy levels derived from electrochemical measurements), the higher polarizability of FBT-EP is expected to play a major role in preventing the percolation of electrons into the lower EML layer when holes are also injected and transported within the EML in biased multilayer OLETs. Evidently, this output is counterintuitive when considering the use of these CPP compounds in vertical-stack optoelectronic devices such as OLEDs. The interplay of a vertical polarization and a horizontal source-drain electric field is of utmost importance when implementing new compounds in OLET devices. 

Overall, FBT-EP showed specific optoelectronic features depending on the device platform, thus proving the versatility of CPP compounds in providing different characteristics from the same chemical structure and solid-state organization.

## 3. Materials and Methods

### 3.1. Device Fabrication and Characterization

OLETs were fabricated on 25 mm × 25 mm transparent glass/ITO substrates covered with a 450 nm thick PMMA layer as gate dielectric according to the literature procedure [63]. OLETs containing the high-k dielectric layer as a gate (Al_2_O_3_) were fabricated on the same 25 mm × 25 mm transparent glass/ITO substrates covered with a 35 nm thick Al_2_O_3_ layer deposited by atomic layer deposition (ALD) technique and 20 nm thick PMMA layer as planarizing layer.

The organic active region consisted of a stacked bilayer of (i) a high-mobility p-type semiconductor C8-BTBT (45 nm) covering the dielectric layer and (ii) a 30 nm thick host− guest EML of Alq_3_ (host) and Pt(OEP) (guest) (doping percentage: 10%) sublimed at 3 and 0.2 Å s^−1^, respectively. The organic trilayer stack was then covered by a 70 nm thick source and drain silver electrodes deposited by thermal evaporation using shadow masks. Both the electrodes and the organic layers were grown at a base pressure of 2.7 × 10^−8^ mbar in a K. J. Lesker chamber directly connected to the nitrogen glove box to prevent sample exposure to air during each step of the device realization. The resulting devices presented the following characteristics: 12 mm channel width (W), 70 µm channel length (L), and 500 µm wide source and drain electrodes. Concerning OLETs containing FBT-EP as EIL and EML, the 60 °C hot solution of FBT-EP was doubly spin-coated at 4000 rpm, with 2000 rpm of acceleration, for 60 s without further treatments. The resulting devices presented the following characteristics: 12 mm channel width (W), 70 μm channel length (L), and 500 μm wide source and drain electrodes. The optoelectronic measurements were performed in an inert atmosphere inside a nitrogen-filled glovebox. The light output was measured at the bottom side of the substrates (i.e., through the gate electrode) with a silicon photodiode (a sensitivity of 0.38 A W^−1^ at 600 nm) directly in contact with the devices to enable the collection of all emitted photons.

The electrical measurements were carried out inside the glovebox using a standard SUSS probe station coupled to a B1500A Agilent semiconductor device analyzer. The field-effect mobility in the saturation regime (μ_sat_) was calculated using the equation I_DS_ = (W/2L)Ci_μsat_(V_G_ − V_T_)^2^, where Ci is the capacitance per unit area of the insulating layer, and V_T_ is the threshold voltage extracted from the square root of the drain current (I_DS_^1/2^) versus gate voltage (V_G_) characteristics. The CLSM images were carried out with a Nikon TE2000 optical microscope, equipped with a 60× objective with 0.70 numerical aperture, connected with a Nikon EZ-C1 confocal scanning head using an excitation wavelength of 405 nm. The optical characterization of OLETs was carried out in air on epoxy resin glass/glass-encapsulated devices to avoid possible degradation of the samples.

### 3.2. Synthesis and Characterization of FBT-EP Polymer

The polymer FBT-EP was synthesized and characterized as previously reported in ref. [46], and the synthesis is reported below for completeness of the results. 

A equimolar mixture of 2,7-Dibromo-9,9-bis(6-bromohexyl)fluorine (0.52 mmol) and 2,1,3-Benzothiadiazole-4,7-bis(boronic acid pinacol ester), with Tetrakis(triphenylphosphine)palladium(0) [Pd(PPh_3_)_4_] (1% mol), tetrabutylammonium bromide (TEBAB) (1%mol) was added to a pre-degassed schlenk, followed by three vacuum/nitrogen cycles. Then, dry toluene (12 mL) and degassed potassium carbonate aqueous solution (2 M, 6 mL) were added. The mixture was stirred at 110 °C. After 3 h, bromobenzene was added to cap the polymer, and 12 h later, phenyl boronic acid was added. The reaction mixture was filtered through a pad of celite and the solvent removed at reduced pressure. The crude was then redissolved in toluene and precipitated in methanol. The polymer was obtained as yellow solid with a yield of 75%.

The obtained polymer was then dissolved in triethyl phosphite under inert atmosphere, and the mixture was stirred at 140 °C overnight. Then, the excess of triethyl phosphite was removed at reduced pressure. The final polymer FBT-EP was obtained as gummy yellow solid in quantitative yield.

Cyclic voltammetry was applied to investigate the electrochemical properties. A glassy carbon coated with FBT-EP was used as the working electrode, and a solution of tetra-n-butylammonium perchlorate (0.1 M Bu4 NClO_4_) in anhydrous acetonitrile was used as the electrolyte. The onset oxidation potential of FBT-EP was 0.97 eV, whereas the reduction potential was −1.72 eV. The highest-occupied molecular orbital (HOMO) and lowest-unoccupied molecular orbital (LUMO) levels were estimated using the equations E_HOMO_ = −(E_ox_ + 4.39  +  0.34) eV and E_LUMO_ = −(E_red_ + 4.39  +  0.34) eV [64], where E_ox_ and E_red_ are the onset reduction and oxidation potentials, respectively, relative to the vacuum scale. The HOMO and LUMO energy levels of FBT-EP were −5.7 eV and −3.01 eV, respectively.

## 4. Conclusions

Among the several optimization strategies used in optoelectronic devices such as OLEDs, OLETs, and OSCs to optimize optoelectronic performance, the use of a conjugated polar polymer as an injection layer in stacked configuration was very promising for improving the injection of charges in the layer where charges recombine. Considering light-emitting transistors, the use of CPP is far from being fully comprehended and optimized in planar field-effect transistor configuration and due to the still-few articles published on this topic. The study reported here focused on the investigation of the charge-injection process in multilayer OLET presenting a functional interface between an electron-transporting semiconductor (i.e., DFH-4T) and an emissive conjugated polar polymer (i.e., FBT-EP). By exploiting the emissive properties and good energy-level match with host matrix in standard EML, we demonstrated that the CPP layer disabled the exciton formation into the EML. The major observable responsible for the deleterious behavior is the possible polarizability of FBT-EP, which probably modifies its LUMO level by local electrical dipole formation and/or orientation at the organic/hybrid interfaces in the multistack. Indeed, we investigated the possible dependence of the modulation of the polarizability of the CPP with the electrostatic potential profile within the active stack in the field-effect device: even by reduction factor of 3 in the drop of the electrostatic potential at the drain electrode by using a high-k dielectric bilayer the emission from EML in the multilayer stacked OLET was inhibited.

In the case of the ambipolar multilayer OLET comprising both hole- and electron-transporting semiconductors, the injection and transport of the holes increased the complexity of the photophysical processes due to the plausible blocking of holes in the EML by the polarization of the CPP layer as the reduction in the collected source-drain current demonstrates. Among the plausible hypotheses capable of explaining the unsuccessful electroluminescence emission in the ambipolar multilayer OLETs with FBT-EP as EIL, the shift of the charge recombination zone into FBT-EP was excluded in virtue of the emissive properties of the compound, while others such as the variation in the energy levels of the orbitals of the CPP in the electron-injection layer due to the polarization-induced dipole generation [59,60] need further detailed investigation to be assessed.

Eventually, the introduction of a luminescent-conjugated polar polymer in an organic stack used as an active region in field-effect transistors allowed the straightforward study of charge- and electric-field-induced processes at the interfaces between organic layers capable of charge accumulation and/or remarkable polarizability. By engineering the active region of prototypal stacked devices for reducing the complexity of the investigated processes, it was possible to better discriminate the route to follow in the realization of effective ambipolar multilayer OLET with respect to the corresponding OLED counterpart. 

## Data Availability

Data are available in the article.

## Supplementary Materials

The following supporting information can be downloaded at: https://www.mdpi.com/article/10.3390/molecules29143295/s1. Figure S1. (a) P-type transfer curves in the saturation regime of the h-OS/EML, h-OS/EIL, and h-OS/EML/EIL OLETs, (b) electroluminescence curves related to h-OS/EML and h-OS/EML/EIL transfer curves, and (c) schematic structures of h-OS/EML, h-OS/EIL, and h-OS/EML/EIL OLETs. Figure S2. (a) Energy levels of the materials used in the OLED. (b) I-V curve of the OLED based on FBT-EP (blue curve) and its respective light emission curve (green curve). On inset the OLED structure.
