# Supplementary material for: A Fluorescent Conjugated Polar Polymer for Probing Charge Injection in Multilayer Organic Light-Emitting Transistors
Source: Molecules. 2024 Jul 12;29(14):3295. doi: 10.3390/molecules29143295 (PMC11279323; doi:10.3390/molecules29143295)
Supplement: Supplementary file 1 [file molecules-29-03295-s001.zip › molecules-3025455-supplementary.pdf]

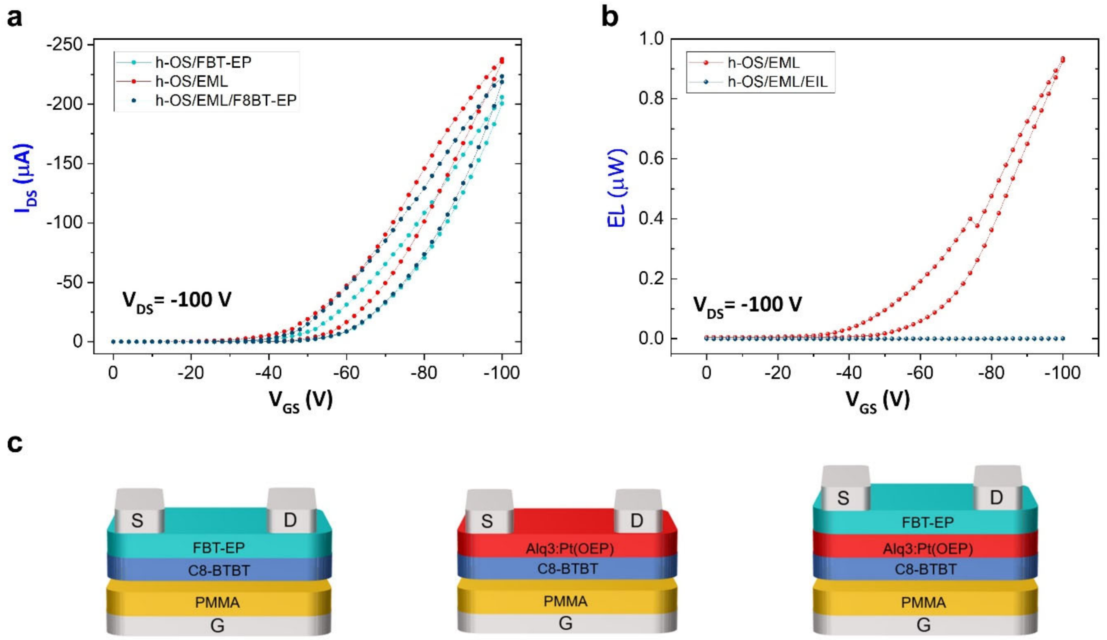


**Figure S1.** (**a**) P-type transfer curves in the saturation regime of the h-OS/EML, h-OS/EIL, and h-OS/EML/EIL OLETs, (**b**) electroluminescence curves related to h-OS/EML and h-OS/EML/EIL transfer curves, and (**c**) schematic structures of h-OS/EML, h-OS/EIL, and h-OS/EML/EIL OLETs.

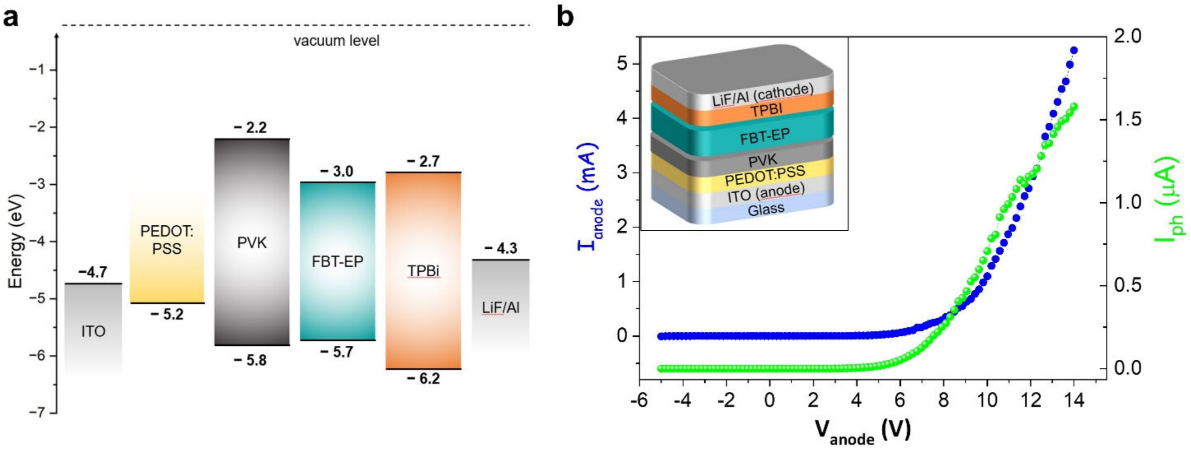


**Figure S2.** (**a**) Energy levels of the materials used in the OLED. (**b**) I-V curve of the OLED based on FBT-EP (blue curve) and its respective light emission curve (green curve). On inset the OLED structure.
